# Cortical folding and the potential for prognostic neuroimaging in schizophrenia

**DOI:** 10.1192/bjp.bp.114.155796

**Published:** 2015-11

**Authors:** Shuixia Guo, Sarina Iwabuchi, Vijender Balain, Jianfeng Feng, Peter Liddle, Lena Palaniyappan

**Affiliations:** **Shuixia Guo**, PhD, College of Mathematics and Computer Science, Key Laboratory of High Performance Computing and Stochastic Information Processing (Ministry of Education of China), Hunan Normal University, Changsha, PR China and Department of Computer Science, University of Warwick, Coventry, UK; **Sarina Iwabuchi**, PhD, Division of Psychiatry & Applied Psychology, University of Nottingham and Centre for Translational Neuroimaging, Institute of Mental Health, Nottingham, UK; **Vijender Balain**, MRCPsych, Penticton Regional Hospital, Penticton, British Columbia, Canada; **Jianfeng Feng**, PhD, Shanghai Center for Mathematical Sciences, Fudan University, Shanghai, PR China and Department of Computer Science, University of Warwick, Coventry, UK; **Peter Liddle**, PhD, Division of Psychiatry & Applied Psychology, University of Nottingham and Centre for Translational Neuroimaging, Institute of Mental Health, Nottingham, UK; **Lena Palaniyappan**, PhD, MRCPsych, Division of Psychiatry & Applied Psychology, University of Nottingham, Centre for Translational Neuroimaging, Institute of Mental Health, Nottingham, UK and Penticton Regional Hospital, Penticton, British Columbia, Canada

## Abstract

In 41 patients with schizophrenia, we used neuroanatomical information derived from structural imaging to identify patients with more severe illness, characterised by high symptom burden, low processing speed, high degree of illness persistence and lower social and occupational functional capacity. Cortical folding, but not thickness or volume, showed a high discriminatory ability in correctly identifying patients with more severe illness.

To date there are no objectives tests that aid prognostic prediction in schizophrenia. Historically, clinical outcomes have improved considerably for medical disorders where severity can be quantified reliably (for example malignancies, asthma). Prognostic prediction, in particular the ability to identify those who will do well in the long term, has proved to be a great challenge in schizophrenia.^1^ Neuroimaging offers the great promise of providing objective measures of clinical utility in managing psychosis.^2^ Recently, the use of multivariate pattern classification in neuroimaging has enabled diagnostic separation at a single patient level.^3^ In this study, we investigated whether this approach can reliably discriminate a patient with less severe illness from one with more severe illness. Given the previous observations that cortical thickness,^4^ folding patterns^5^ and grey matter volume^6^ relate to prognosis in schizophrenia, we employed these surface-based morphometric measures to identify illness severity.

## Method

A sample of 41 patients with a DSM-IV diagnosis^7^ of schizophrenia or schizoaffective disorder was recruited for this study. This sample is described in detail in our previous studies.^8,9^ The clinical severity was quantified using a composite index derived from symptom burden, functional ability, cognition and persistence of illness as described in our previous work^8^ and in online supplement DS1. Using this severity index, 20 participants were classified as having a high severity of illness with the remaining 21 having a low severity of illness. The clinical and demographic characteristics of the two groups are presented in online supplement DS1 and Table DS1.

Structural magnetic resonance imaging scans obtained from the participants were processed using Freesurfer (5.1.0) (http://surfer.nmr.mgh.harvard.edu/) as previously described.^10^ Reconstructed surfaces were inspected for topological defects and edited in accordance with our previous work^11^ by a single rater (L.P.) masked to the severity status at the time of surface editing. Cortical folding was measured using local gyrification index proposed by Schaer *et al*.^12^ Cortical thickness was estimated using the standard procedures described by Fischl & Dale.^13^ The reconstructed brain surfaces were parcellated using the Destrieux atlas to provide 148 brain regions based on sulcogyral boundaries described by Duvernoy.^14^ For each metric, these 148 values were used as features in the classifier.

We used a linear support vector machine (SVM) proposed by Cortes & Vapnik^15^ and implemented by the libsvm toolkit (http://www.csie.ntu.edu.tw/~cjlin/libsvm/). SVM is a statistical discrimination procedure that finds a linear separation surface in the high-dimensional multivariate feature space that maximally separates the training data into two classes as specified by the pre-assigned labels (in this case, high and low severity groups). Based on this separation, the class membership of a new participant (test data) can be predicted, and the accuracy of these predictions quantified. Further details are given in online supplement DS1. We computed test performance measures and diagnostic odds ratio using a leave-one-subject-out (LOSO) cross validation. The statistical significance of these measures was determined using permutation testing (*n* = 1000 permutations).

## Results

Table 1 displays the accuracy of the classification and the most significant predictors of the best performing classifier. Given that gender and parental socioeconomic status differed between the two groups, we regressed out the variance explained by these two variables, and repeated the SVM analysis. Our results continued to show a superior, statistically significant accuracy for regional gyrification but not for thickness or volume (online supplement DS1, Fig. DS1 and Tables DS3 and DS4).

**Table 1:** Test performance measures of morphometric multivariate pattern classifiers^a^

Region	Accuracy, % (*P*)	Sensitivity, %	Specificity, %	Likelihood ratio (positive and negative)	Diagnostic odds ratio
Thickness	65.9 (0.07)	70	61.9	1.84, 0.48	3.79
Gyrification	73.2 (0.004)	60	85.7	4.20, 0.47	9.00
Volume	51.2 (0.5)	0	100	–	–

a. *P*-values are not corrected for multiple comparisons.

## Discussion

To our knowledge, this is the first study to investigate the prospect of exploiting multivariate neuroanatomical information to predict clinical severity of schizophrenia at the individual level. Using a classifier based on the features of cortical folding, we can identify the degree of illness severity in medicated, community-living patients with clinically stable schizophrenia. This predictive ability appears to be a unique feature of folding patterns, as the classifiers based on thickness and volume do not perform significantly above chance when separating high and low illness severity groups. Furthermore, patients with greater illness severity had reduced cortical folding in most brain regions, suggesting that a distributed defect in cortical morphology influences prognosis. Although the accuracy achieved by the gyrification-based classifier is statistically significant, the performance of this classifier is considerably weaker when compared with the multivariate neuroanatomical classifiers tested in the separation of healthy controls from patients with schizophrenia.^16^ There may be several reasons for this disparity. The use of median split to divide the sample into high and low severity could have contributed to the lack of strong between-groups discriminative features With larger samples, extreme prognostic groups (lying on either end of the severity continuum) could be used for training the classifier and improve the accuracy. It is worth noting that in clinical practice, it is rarely necessary to apply a test to differentiate a patient with schizophrenia from a healthy control. The classification of a patient with schizophrenia from a healthy control can be done clinically with a high degree of confidence, thus even a high-performance neuroimaging test will have limited clinical utility in this context. On the other hand, at present there are no reliable means of predicting prognostic group membership; even a test that increases the likelihood of identifying prognostic grouping to a moderate extent, could be of significant benefit to patients and clinicians.

We quantified illness severity on the basis of a number of variables; this approach offered a multidomain metric that reflected symptom burden across the three syndromes of schizophrenia, a cognitive function that is most prominently affected in schizophrenia i.e. processing speed, social and functional performance and persistence of illness. Nevertheless, various other metrics relevant for the assessment of severity (such as Clinical Global Impression, quality of life scales, self-rated recovery measures or assessments of daily living) were not collected in this study. Furthermore, from this cross- sectional study it is not possible to extrapolate whether a gyrification-based classifier applied at illness onset could prospectively predict later severity. Nevertheless, when compared with cortical thickness and volume, gyrification has been shown to be relatively stable during adult life.^17^ In addition, a large degree of variance in the cortical folding patterns relates to neurodevelopmental integrity during the fetal or early neonatal period.^18^ Taken together, these observations suggest that the burden of neurodevelopmental abnormalities in a patient with schizophrenia could be a potential influence on illness severity.

Our results provide preliminary evidence for the utility of cortical folding in single participant-level prognostic imaging in schizophrenia. With larger validation studies that combine high-yield clinical prognostic indicators with gyrification metrics, the predictive value can be further improved, enabling an objective grading of outcome in the management of schizophrenia. This has the promise of assisting targeted service delivery and making personalised recommendations with regard to the required duration of antipsychotic treatment. Most importantly, this approach can be refined to provide accurate information on the chances of a satisfactory clinical recovery and thus potentially empower patients by addressing the uncertainty that surrounds prognosis in psychotic disorders.
